# CDX2: A Prognostic Marker in Metastatic Colorectal Cancer Defining a Better *BRAF* Mutated and a Worse *KRAS* Mutated Subgroup

**DOI:** 10.3389/fonc.2020.00008

**Published:** 2020-02-11

**Authors:** Kristine Aasebø, Anca Dragomir, Magnus Sundström, Artur Mezheyeuski, Per-Henrik Edqvist, Geir Egil Eide, Fredrik Ponten, Per Pfeiffer, Bengt Glimelius, Halfdan Sorbye

**Affiliations:** ^1^Department of Clinical Science, University of Bergen, Bergen, Norway; ^2^Department of Pathology, Uppsala University Hospital, Uppsala, Sweden; ^3^Department of Immunology, Genetics and Pathology, Uppsala University, Uppsala, Sweden; ^4^Department of Global Public Health and Primary Care, Lifestyle Epidemiology Group, University of Bergen, Bergen, Norway; ^5^Centre for Clinical Research, Haukeland University Hospital, Bergen, Norway; ^6^Science for Life Laboratory, Uppsala University, Uppsala, Sweden; ^7^Department of Oncology, Odense University Hospital, Odense, Denmark; ^8^Department of Oncology, Haukeland University Hospital, Bergen, Norway

**Keywords:** caudal type homeobox transcription factor, CDX2, colorectal cancer, metastatic disease, stage 4 colorectal cancer, prognosis, population based

## Abstract

**Background:** Survival of metastatic colorectal cancer (mCRC) patients has improved, but mainly for trial patients. New predictive and prognostic biomarkers validated in the general mCRC population are needed. Caudal-type homeobox 2 (CDX2) is an intestine-specific transcription factor with potential prognostic and predictive effect, but the importance in mCRC has not been fully investigated.

**Methods:** Immunohistochemistry analysis of CDX2 was performed in a Scandinavian population-based cohort of mCRC (*n* = 796). Frequency, clinical and tumor characteristics, response rate, progression-free survival, and overall survival (OS) were estimated.

**Results:** Loss of CDX2 expression was found in 87 (19%) of 452 stained cases, in 53% if *BRAF* mutated (*BRAF*mut) and in 9% if *KRAS* mutated (*KRAS*mut). CDX2 loss was associated with microsatellite instability, *BRAF*mut, and poor differentiation and inversely associated with *KRAS*mut. Patients with CDX2 loss received less first-line (53 vs. 64%, *p* = 0.050) and second-line (23 vs. 39%, *p* = 0.006) chemotherapy and secondary surgery (1 vs. 9%, *p* = 0.019). Median progression-free survival and OS for patients given first-line combination chemotherapy was 4 and 10 months if CDX2 loss vs. 9 and 24 months if CDX2 expressed (*p* = 0.001, *p* < 0.001). Immediate progression on first-line combination chemotherapy was seen in 35% of patients with CDX2 loss vs. 10% if CDX2 expressed (*p* = 0.003). Median OS in patients with *BRAF*mut or *KRAS*mut and CDX2 expressed in tumor (both 21 months) was comparable to wild-type patients (27 months). However, if CDX2 loss, median OS was only 8 and 11 months in *BRAF*mut and *KRAS*mut cases, respectively, and 10 months in double wild-type patients. In multivariate analysis, CDX2 loss (hazard ratio: 1.50, *p* = 0.027) and *BRAF*mut (hazard ratio: 1.62, *p* = 0.012) were independent poor prognostic markers for OS.

**Conclusion:** In a population-based cohort of mCRC patients, CDX2 loss is an independent poor prognostic marker. Expression of CDX2 defines a new subgroup of *BRAF*mut cases with a much better prognosis. Loss of CDX2 defines a small group of *KRAS*mut cases with a worse prognosis. Patients with CDX2 loss receive less palliative chemotherapy with less benefit and rarely reach secondary surgery.

## Introduction

Colorectal cancer (CRC) is one of the major cancer types worldwide. Globally, there are 1.4 million new cases and 0.7 million deaths in 2012 (1, 2). Approximately 25% of patients present with metastatic CRC (mCRC) at diagnosis, and another 20% will eventually develop metastasis. Despite progress over the past decades, with median overall survival (OS) up to 30 months in clinical trials, prognosis for patients in population-based cohorts is still poor with a median OS of 10–15 months (3, 4). Patients included in clinical trials are highly selected with, for instance, better performance status, younger age, and less or no comorbidity, and cannot be compared to the general mCRC patients. There is a need for predictive and prognostic markers validated in population-based cohorts to guide treatment selection and improve survival for mCRC patients.

Caudal-type homeobox 2 (CDX2) is an intestine-specific transcription factor and one of the most sensitive and specific markers of intestinal differentiation (5). Immunohistochemistry (IHC) analysis for CDX2 is implemented in the clinical diagnostics as a biomarker for intestinal differentiation in tumors of unknown origin. It is deregulated in a subset of patients with CRC, and downregulation has been associated with poor prognosis (6–13). Loss of CDX2 expression has also been associated with other poor prognostic features such as advanced stages, poor differentiation, *BRAF* mutation (*BRAF*mut), and microsatellite instability (MSI) (12, 14, 15). The negative prognostic effect of CDX2 could therefore be related to the known associations between these features and poor survival. No previous studies have fully explored if the negative prognostic effect of CDX2 loss in mCRC could be confounded by these associations. Two recent retrospective studies have reported CDX2 loss as a predictive biomarker for treatment benefit of chemotherapy in stage II (7) and stage III (8) CRC. This has so far not been demonstrated in mCRC (8, 9, 11). Recently, CDX2 loss was reported to be an independent negative prognostic marker in mCRC patients undergoing curative liver metastasis resection, indicating CDX2 loss as a potential biomarker to identify patients with limited benefit from surgery (11). It is therefore important to know the proper frequency of CDX2 loss and its relations to outcome in unselected mCRC patients. The aim of this study was to determine the prevalence, prognostic, and predictive effect of CDX2 loss in unselected patients of mCRC in relation to tumor differentiation, *BRAF, KRAS*, and MSI status.

## Methods

### Patient Cohort

**Figure 1:**
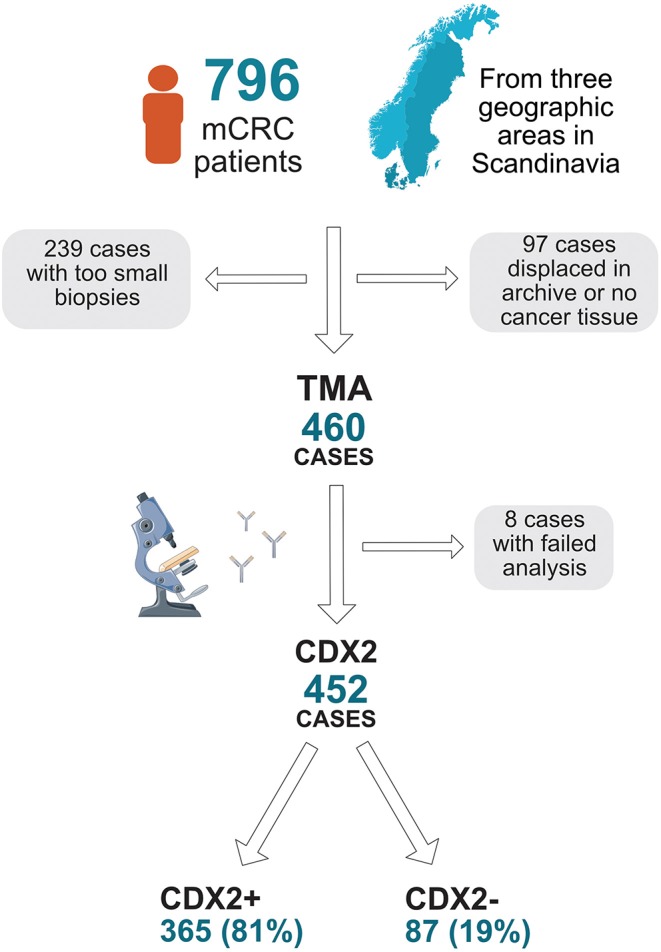
Flow chart describing collection of tumor blocks, tissue microarray (TMA), and availability of CDX2 status in a population-based Scandinavian cohort of metastatic colorectal cancer.

A prospectively collected cohort was established of all non-resectable mCRC patients referred to the oncology units of three regional hospitals in Scandinavia: Odense University Hospital (Denmark) (*n* = 325), Uppsala University Hospital (Sweden) (*n* = 155), and Haukeland University Hospital (Norway) (*n* = 316) during 2003–2006 with last follow-up in 2014. These hospitals cover all oncology treatment in their region. Cases not referred in the region were identified through the national (Norway and Sweden) and regional (Denmark) cancer registries (*n* = 49). The cohort consists of 796 patients (Figure 1).

### Tissue Retrieval and Tissue Microarray Generation

Paraffin-embedded tissue blocks were retrieved from the primary tumor in the majority of cases or from a metastatic lesion (six cases), and corresponding hematoxylin–eosin stained glass slides were examined. Tissue microarray (TMA) generation had been performed previously in 460 (58%) cases (16) according to standards used in the Human Protein Atlas (17), with two 1-mm diameter tumor cores extracted per patient. TMA was generated from tissue blocks from surgical resection of primary tumor in 419 of 460 (91%) TMA cases, the remaining 41 from biopsies (35 cases from primary tumor and 6 cases from metastatic lesion).

### Tumor Analyses

**Figure 2:**
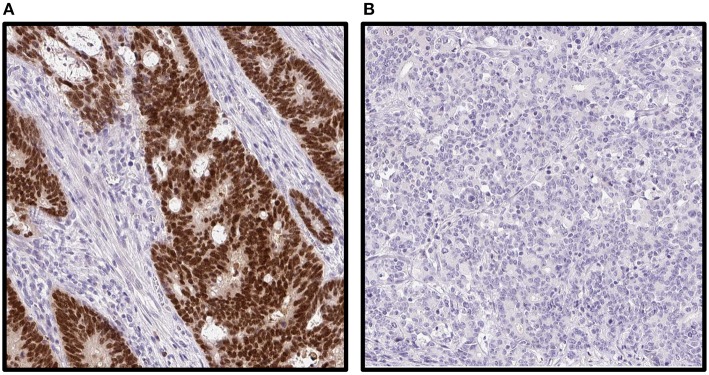
Immunohistochemical staining images of caudal-type homeobox 2 (CDX2) on tumor tissue microarray in a population-based Scandinavian cohort of metastatic colorectal cancer patients. **(A)** Strong staining in all cells. **(B)** Completely negative staining.

Results on gene analysis of *KRAS, BRAF*, and MSI in *BRAF*mut tumors and IHC analysis on *BRAF* and MMR were available and performed as described previously (16, 18). IHC for CDX2 was performed for all patients included in the TMA cohort (*n* = 460) using a mouse-monoclonal antibody, #NCL-CDX2, from Leica Biosystems (formerly Novocastra), diluted 1:50. Automated IHC was performed using an Autostainer 480 instrument (Thermo Fischer Scientific, Waltham, MA, United States), with diaminobenzidine (Thermo Fisher Scientific) as chromogen. High-resolution images of the IHC staining were obtained by scanning with an Aperio AT2 slide scanner (Aperio, Vista, CA, United States) at 200× magnification. Semiquantitative assessment of immunoreactivity in all tumor cells was assessed independently by two pathologists (AD, FP) without knowledge of clinicopathological data. Annotation discrepancies were re-evaluated to reach consensus. Immunoreactivity was scored for nucleus on a four-tier intensity scale (1 = negative, 2 = weak, 3 = moderate, or 4 = strong), and the estimated fraction of stained tumor cells was denoted as 1 (0–1%), 2 (2–10%), 3 (11–25%), 4 (26–50%), 5 (51–75%), and 6 (>75%) (Figure 2). Loss of CDX2 expression (CDX2 loss) was defined as tumors with nuclear fraction staining <10% regardless of intensity, as recommended in the interpretation of IHC of tumor markers in CRC (19). This cutoff was chosen according to previous literature, and the distribution of expression across the cohort. CDX2 expression was defined as tumors with nuclear fraction staining >10% regardless of intensity. CDX2 status was evaluable in 452 cases (Figure 1).

### Statistics

Exact chi-square test was used for group comparisons. Multiple binary logistic regression was used for dichotomous outcome variables, and results are reported as odds ratios (ORs) with 95% confidence intervals (CIs). OS was the interval from the date of metastatic disease to the date of death and censored if the patient was alive on February 4th, 2014. Progression-free survival (PFS) was the interval from the date of first administration of chemotherapy to the date of progression (on CT scan) or death and censored if the patient was alive without progression on February 4th, 2014. OS and PFS were analyzed using the Kaplan–Meier method with log rank test and Cox multiple regression. Cox regression was performed with backward stepwise selection of covariates to the final model. At the first step, we included all relevant covariates. From this model, we removed the variable with the largest *p*-value. In the second step, we removed the covariate with largest *p* > 0.05 among the remaining variables from the first step. The process continued until all remaining variables were significant at level 0.05, and a final model was obtained. Results are reported as hazard ratios (HRs) and 95% CIs. All analyses were performed with the statistical program SPSS v22. All statistical tests were two-tailed using 5% significance level.

## Results

### Study Population

**Figure 3:**
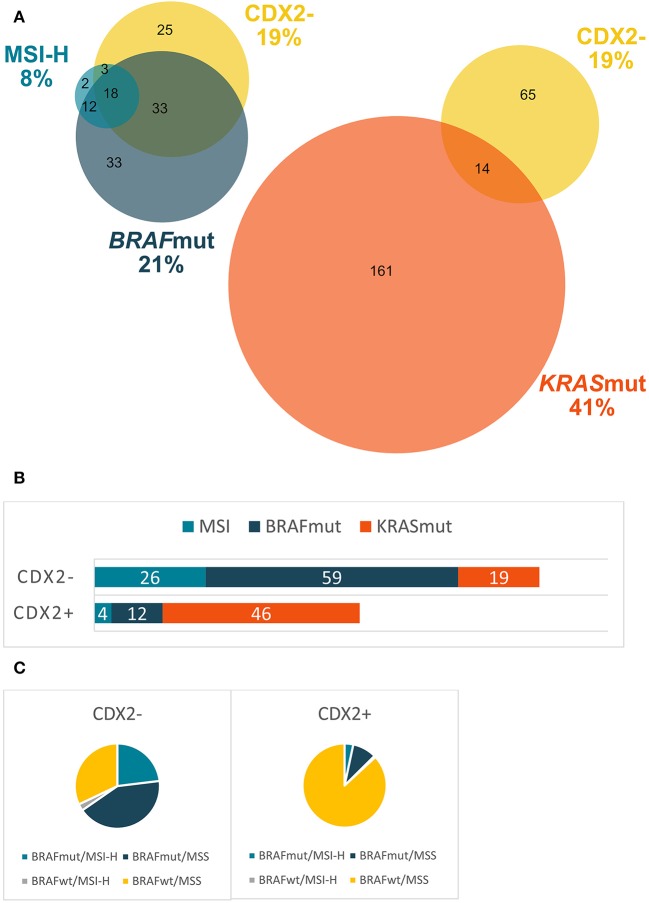
Distribution of different molecular markers in a population-based Scandinavian cohort of metastatic colorectal cancer. **(A)** Frequency (%) of *BRAF*, microsatellite instability (MSI), *KRAS*, and caudal-type homeobox 2 (CDX2) status and their interrelations. **(B)** Frequency (%) of *BRAF*, MSI-H, and *KRAS* mutations in tumors with CDX2 loss and CDX2 expression. **(C)** *BRAF*/MSI subgroups in tumors with CDX2 loss and CDX2 expression. *KRAS*mut, *KRAS* mutation; *BRAF*mut, *BRAF* mutation; MSI-H, microsatellite instable high; MSS, microsatellite stable; double wild type, wild-type *KRAS* and *BRAF*; CDX2–, CDX2 loss; CDX2+, CDX2 expression.

Of the 796 patients included, 460 patients had TMA generated; reasons for no TMA were too small biopsies with no resection of primary tumor performed (*n* = 239) and missing or no cancer tissue (*n* = 97) (Figure 1). Cases with lacking TMA was evenly distributed between the three regions of inclusion (45% from Haukeland, 36% from Uppsala, and 43% from Odense University Hospital, *p* = 0.190). For patients with CDX2 status available, median OS was 11 months (9.4, 12.6) for all patients (*n* = 452), 18 months (15.0, 21.0) if given first-line chemotherapy (*n* = 281) and 21 months (17.1, 24.9) if given first-line combination chemotherapy (*n* = 217). As first-line treatment, 52 patients received irinotecan-based and 168 oxaliplatin-based combination chemotherapy, and 57 received 5-fluorouracil monotherapy. Twenty-one had bevacizumab and 20 had cetuximab combination treatment. Combination chemotherapy was mainly doublet; only three patients received triplet chemotherapy. There was no significant difference in treatment schedules given between patients with loss or expression of CDX2 (Table S1). For patients with CDX2 status available, 21% had a *BRAF* V600E mutation (*BRAF*mut), 41% a *KRAS* mutation (*KRAS*mut), 8% were MSI high (MSI-H), and 38% double (*KRAS* and *BRAF*) wild-type tumor (Table 1, Figure 3A).

**Table 1:** Patient characteristics according to caudal-type homeobox 2 status in a population-based cohort of metastatic colorectal cancer with CDX2 status available (*n* = 452).

Characteristics^*^	**Patients with CDX2 status**	**Missing**	**CDX2–**	**CDX2+**	**CDX2– vs. CDX2+ *p*-value**
Total number (%)	452		87 (19)	365 (81)	
Age in years, median (95% CI)	70 (68.0, 70.2)		70 (66.5, 71.5)	70 (67.8, 70.3)	0.957
Age > 75 years, *n* (%)	155 (34)		31 (36)	124 (34)	0.802
Female, *n* (%)	229 (51)		50 (58)	179 (49)	0.189
PS WHO > 1, *n* (%)	152 (34)		37 (43)	115 (32)	0.058
Right sided, *n* (%)	177 (40)	7	52 (60)	125 (35)	<0.001
Liver metastases, *n* (%)	287 (64)		49 (56)	238 (65)	0.137
Liver only, *n* (%)	118 (26)		14 (16)	104 (29)	0.018
Lung metastases, *n* (%)	113 (25)		14 (16)	99 (27)	0.038
Lymph node metastases, *n* (%)	131 (29)		37 (43)	94 (26)	0.003
Peritoneal metastases, *n* (%)	88 (20)		23 (26)	65 (18)	0.072
>1 metastatic site, *n* (%)	262 (58)		55 (63)	207 (57)	0.280
Synchronous metastases, *n* (%)	244 (54)		54 (62)	190 (52)	0.095
ALP high, *n* (%)	222 (56)	55	43 (60)	179 (55)	0.513
Primary tumor resected, *n* (%)	414 (92)		80 (92)	331 (92)	1.000
Tumor grade 1, *n* (%)	55 (13)	15	7 (8)	48 (14)	<0.001
2, *n* (%)	288 (66)		39 (47)	249 (70)	
3, *n* (%)	94 (22)		37 (45)	57 (16)	
*KRAS* mutation, *n* (%)	179 (41)	15	16 (19)	163 (46)	<0.001
*BRAF* mutation, *n* (%)	96 (21)	9	51 (59)	45 (12)	<0.001
Double wild type, *n* (%)	164 (38)	15	18 (21)	146 (41)	0.001
MSI-H, *n* (%)	35 (8)	11	21 (26)	14 (4)	<0.001
*BRAF*mut/MSI-H, *n* (%)	30 (7)	16	18 (23)	12 (3)	<0.001
*BRAF*mut/MSS, *n* (%)	66 (15)		33 (42)	33 (9)	
*BRAF*wt/MSI-H, *n* (%)	5 (1)		3 (4)	2 (1)	
*BRAF*wt/MSS, *n* (%)	336 (77)		25 (32)	311 (87)	
Curative metastasis surgery, *n* (%)	33 (7)	1	1 (1)	32 (9)	0.019
First-line chemotherapy, *n* (%)	281 (62)		46 (53)	235 (64)	0.050
Combination, *n* (%)	217 (77)		34 (74)	183 (78)	0.567
Monotherapy, *n* (%)	64 (23)		12 (26)	52 (22)	
Second-line chemotherapy, *n* (%)	162 (36)	1	20 (23)	142 (39)	0.006
Third-line chemotherapy, *n* (%)	72 (16)	1	2 (2)	70 (19)	<0.001
BSC only, *n* (%)	170 (38)		41 (47)	129 (35)	0.049

*CDX2–, CDX2 loss; CDX2+, CDX2 expression; MSI-H, microsatellite instable high; MSS, microsatellite stable; PS ECOG, performance status score developed by Eastern Cooperative Oncology Group; Right sided, site of colon cancer in ascending colon and transversum; Left sided, site of colon cancer in descending colon, sigmoid, and rectum; Metastases, at time of diagnosis of metastatic disease; Synchronous metastases, within 6 months after initial diagnose; ALP high, alkaline phosphatase >105 U/L; Double wild type, both BRAF and KRAS wild type; BRAFmut, BRAF mutated; BRAFwt, BRAF wild type; BSC, best supportive care; p-value, chi-square test except for age (t-test)*.

* *Percentage is calculated without missing values. Owing to rounding, not all percentages are 100 in total*.

### Patient Characteristics and Treatment

Eighty-seven (19%) of 452 patients had CDX2 loss (Figure 3A). Frequency of CDX2 loss was similar (20%) among the subgroup of elderly patients (>75 years). In patients with MSI-H, *BRAF*mut and *KRAS*mut tumors, 58, 53, and 9% had CDX2 loss, respectively. A Venn diagram (Figure 3A) illustrates the frequency of CDX2 loss, MSI-H, *BRAF*mut, and *KRAS*mut, and their interrelations. CDX2 loss was associated with right-sided primary tumor, poor differentiation, MSI-H, *BRAF*mut, and *KRAS* wild type (*KRAS*wt) (Table 1, Figure 3B). In subgroup analyses, cases with CDX2 loss were mostly *BRAF*mut/MSS (42%), and cases with CDX2 expression were mostly *BRAF*wt/MSS (87%) (Figure 3C). Patients with CDX2 loss had less often lung metastases and liver-only disease, but more often distant lymph node metastases (Table 1). In multiple logistic regression, *BRAF*mut and poor differentiation were significantly correlated to CDX2 loss (Table S2). Patients with CDX2 loss received less first- (53 vs. 64%, *p* = 0.050) and second-line chemotherapy (23 vs. 39%, *p* = 0.006) and rarely had secondary surgery (1 vs. 9%, *p* = 0.019) compared to patients with CDX2 expressed.

### Survival and Response

**Figure 4:**
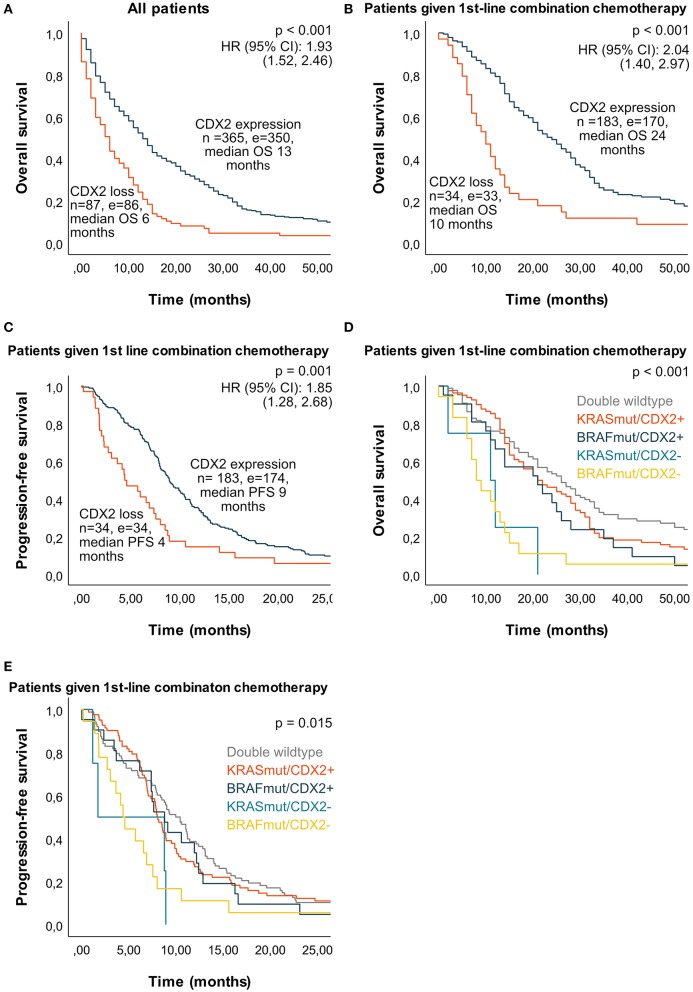
Median overall survival (OS) and progression-free survival (PFS) in a population-based Scandinavian cohort of metastatic colorectal cancer according to tumor molecular alterations. Kaplan–Meier curves were calculated with log-rank test for *p* value and univariate Cox regression for HR and 95% CI. **(A)** Median OS for all patients according to CDX2 status. **(B)** Median OS according to CDX2 status for patients given first-line combination chemotherapy. **(C)** Median PFS according to CDX2 status for patients given first-line combination chemotherapy. **(D)** Median OS according to tumor molecular alterations in patients given first-line combination chemotherapy: double wild type 26 months (*n* = 88, *e* = 80), *KRAS*mut/CDX2 expressed 21 months (*n* = 82, *e* = 77), *BRAF*mut/CDX2 expressed 21 months (*n* = 21, *e* = 20), *KRAS*mut/CDX2 loss 11 months (*n* = 4, *e* = 4), *BRAF*mut/CDX2 loss 8 months (*n* = 18, *e* = 18). **(E)** Median PFS according to tumor molecular alterations in patients given first-line combination chemotherapy: double wild type 10 months (*n* = 88, *e* = 85), *KRAS*mut/CDX2 expressed 8 months (*n* = 83, *e* = 78), *BRAF*mut/CDX2 expressed 9 months (*n* = 21, *e* = 20), *KRAS*mut/CDX2 loss 2 months (*n* = 4, *e* = 4), *BRAF*mut/CDX2 loss 4 months (*n* = 18, *e* = 18). *n*, number; *e*, events; double wild type: *KRAS* and *BRAF* wild type; *BRAFmut, BRAF* mutation; *KRASmut, KRAS* mutation; *HR*, hazard ratio; CI, confidence interval; CDX2–, CDX2 loss; CDX2+, CDX2 expression.

**Figure 5:**
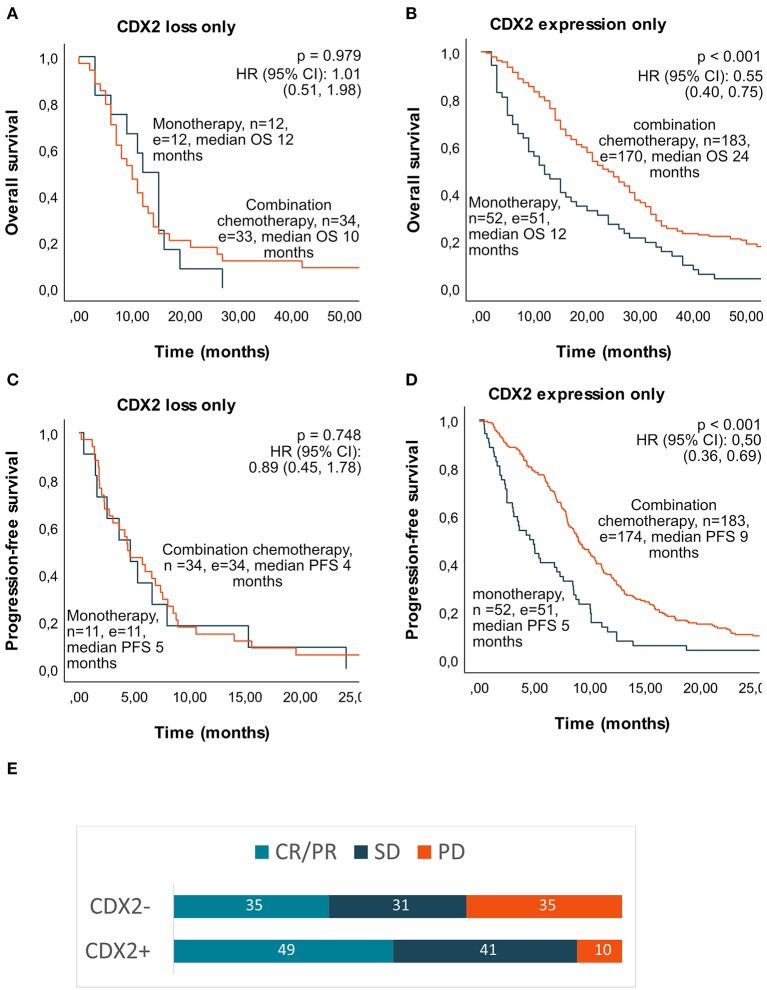
Median overall survival (OS), progression-free survival (PFS), and response rate if given first-line mono- or combination chemotherapy according to tumor CDX2 status in a population-based Scandinavian cohort of metastatic colorectal cancer. Kaplan–Meier curves were calculated with log-rank test for *p*-value and univariate Cox regression for HR and 95% CI. **(A)** Median OS in patients with CDX2 loss. **(B)** Median OS in patients with CDX2 expressed. **(C)** Median PFS in patients with CDX2 loss. **(D)** Median PFS in patients with CDX2 expressed. **(E)** Response rate (%) after first-line combination chemotherapy according to CDX2 status. *n*, number; *e*, events; HR, hazard ratio; CI, confidence interval; CR, complete response; PR, partial response; SD, stable disease; PD, progressive disease.

Median OS in the whole cohort (untreated and treated with chemotherapy) was 6 months if tumor had CDX2 loss and 13 months if tumor showed CDX2 expression (*p* < 0.001). For patients given first-line chemotherapy, median OS was 11 months if CDX2 loss (*n* = 46) and 21 months if CDX2 expressed (*n* = 235) (*p* < 0.001). For patients given first-line combination chemotherapy, median OS was 10 months if CDX2 loss and 24 months if CDX2 expressed (*p* < 0.001) (Figure 4). Median PFS after first-line chemotherapy was 5 vs. 8 months (*p* = 0.003) in patients with CDX2 loss vs. expression and 4 vs. 9 months (*p* = 0.001) if given first-line combination chemotherapy (Figure 4). We did not observe any survival benefit of combination chemotherapy compared to monotherapy in first-line treatment of patients with tumors demonstrating CDX2 loss (median OS 10 vs. 12 months, *p* = 0.979 and median PFS 5 vs. 4 months, *p* = 0.742), but this was evident in patients with tumors demonstrating CDX2 expression (median OS 12 vs. 24 months, *p* < 0.001 and median PFS 5 vs. 9 months *p* < 0.001) (Figure 5). For patients given first-line combination chemotherapy with response registered (*n* = 194 of 217), objective response rate was 35% if the tumor showed CDX2 loss and 49% if CDX2 was expressed. Immediate disease progression was seen in 35% of patients with CDX2 loss compared to 10% with CDX2 expressed (Table S3, Figure 5E).

In further subgroup survival analyses, we selected patients given first-line combination chemotherapy to eradicate potential treatment selection bias (Figure 4D). In patients with double (*KRAS* and *BRAF*) wild-type tumor, median OS was 10 months if CDX2 loss (*n* = 10) compared to 27 months if CDX2 expressed (*n* = 77) (*p* = 0.576). Patients with *KRAS*mut tumor had median OS of 11 months if CDX2 loss (*n* = 4) compared to 21 months if CDX2 expressed (*n* = 83) (*p* = 0.007). Patients with *BRAF*mut tumors had median OS of 8 months if CDX2 loss (*n* = 18) compared to 21 months if CDX2 expressed (*n* = 21) (*p* = 0.008). Owing to selection of patients treated with combination chemotherapy, we had few patients in some subgroups, particularly of cases with *KRAS*mut and CDX2 loss. However, when analyzing all *KRAS*mut cases, regardless of treatment, median OS was only 2 months if CDX2 loss (*n* = 16) compared to 13 months if CDX2 expressed (*n* = 163) (*p* < 0.001). The negative prognostic effect of CDX2 loss was also significant when analyzing all *BRAF*mut cases, with 6 months median OS if CDX2 loss (*n* = 51) compared to 10 months if CDX2 expressed (*n* = 45) (*p* = 0.008) (Figure S1). In patients treated with first-line chemotherapy, the negative prognostic potential of CDX2 loss was seen regardless of *BRAF, KRAS*, and MSI status (Figure S2), however with few patients in some of the subgroups. CDX2 loss was a poor prognostic marker regardless of tumor side (left-sided 12 vs. 25 months and right-sided 8 vs. 22 months median OS, *p* < 0.001) and tumor grade (poorly differentiated 6 vs. 20 months, *p* < 0.001 and well/moderately differentiated 14 vs. 25 months median OS, *p* = 0.002) in patients treated with first-line combination chemotherapy. Elderly given first-line chemotherapy had a median OS of 9 months if CDX2 loss compared to 15 months if CDX2 expressed (*p* = 0.048).

In multiple Cox regression analyses, with known prognostic factors for mCRC survival, both CDX2 loss and *BRAF*mut (among others) were statistically significant associated with reduced OS. CDX2 loss, MSI-H, *BRAF*mut, and *KRAS*mut (among others) with shorter PFS (Table 2).

**Table 2:** Results from multiple Cox regression of overall survival and progression-free survival in a population-based Scandinavian cohort of metastatic colorectal cancer patients.

	Overall survival^*^ **(*****n*** **=** **357**, ***e*** **=** **341)**			**Progression-free survival after first-line chemotherapy (*****n*** **=** **245**, ***e*** **=** **235)**		
**Variable**	**HR**	**95% CI**	***p*-value**	**HR**	**95% CI**	***p*-value**
Female	0.85	(0.68, 1.03)	0.148	0.94	(0.72, 1.23)	0.655
AGE >75 years	1.04	(0.74, 1.46)	0.844	1.59	(1.02, 2.46)	0.039
PS WHO > 1	1.78	(1.36, 2.34)	<0.001	2.02	(1.39, 2.93)	<0.001
Right-sided tumor	1.06	(0.82, 1.37)	0.662	0.75	(0.55, 1.04)	0.081
Tumor grade 3	1.66	(1.22, 2.25)	0.001	1.55	(1.08, 2.23)	0.019
Primary tumor resected	1.14	(0.65, 2.00)	0.657	1.65	(0.92, 2.97)	0.095
Synchronous metastases	0.70	(0.55, 0.88)	0.002	0.75	(0.56, 1.01)	0.056
>1 organ metastases	1.34	(1.02, 1.76)	0.038	1.47	(1.00, 2.15)	0.049
Liver only	1.19	(0.87, 1.62)	0.271	1.40	(0.91, 2.16)	0.125
Curative metastasis surgery	0.32	(0.20, 0.52)	<0.001	0.38	(0.24, 0.62)	<0.001
ALP high	1.98	(1.55, 2.54)	<0.001	1.55	(1.15, 2.09)	0.004
First-line chemotherapy	0.37	(0.25, 0.53)	<0.001	n.i.		
MSI-H	1.38	(0.83, 2.30)	0.212	2.08	(1.06, 4.08)	0.032
*KRAS* mutation	1.25	(0.97, 1.62)	0.088	1.62	(1.17, 2.23)	0.003
*BRAF* mutation	1.62	(1.11, 2.35)	0.012	1.63	(1.04, 2.55)	0.032
CDX2 loss	1.50	(1.05, 2.15)	0.027	1.54	(1.00, 2.35)	0.049

*n, number of patients; e, number of events; HR, hazard ratio; CI, confidence interval; p value, from likelihood ratio test; PS ECOG, performance status score developed by Eastern Cooperative Oncology Group; Right-sided tumor, site of colon cancer in ascending colon and transversum; Synchronous metastases, within 6 months after initial diagnose; ALP high, alkaline phosphatase > 105 U/L; MSI-H, microsatellite instable high; n.i., not included*.

* *CEA > 4 and LDH high was also statistically significant when included in the multiple regression model but were excluded from the analysis due to many missing values*.

## Discussion

This is the largest study of incidence of CDX2 loss and its correlation to treatment and survival in a population-based cohort of mCRC and the first study that also corrects for the prognostic markers MSI, *BRAF*, and *KRAS* status in the analyses. The generally poor survival in our cohort is comparable to Scandinavian cancer registries (3) and the American SEER database during the same time period (20), reflecting our unselected population of mCRC. A recent study confirms the low median OS of mCRC patients in the general population compared to phase III trial results (4). Our real-world data shows that mCRC patients who have non-resectable metastatic disease with CDX2 loss have a worse prognosis, receive less first- and second-line chemotherapy with less benefit and rarely receive secondary surgery.

The incidence of CDX2 loss was 19%, comparable to previous published results on stage IV CRC patients in population-based cohort studies (8, 15). Others have reported lower frequency of CDX2 loss, but most of these studies have very few patients with stage IV disease (21, 22). Two recent mCRC studies showed only 3–6% CDX2 loss, probably reflecting the selection of patients with better prognosis into clinical trials (10) and patients treated at the Mayo Clinic (9) compared to our population-based cohort. CDX2 loss was significantly associated with poor prognostic markers such as MSI-H, *BRAF*mut, right-sided tumors, and poor differentiation, in accordance with previous reports (8, 12, 15). In our study, we demonstrate that CDX2 loss is an independent negative prognostic tumor marker, even after correcting for these known prognostic factors.

*BRAF*mut is considered the clinically most important negative prognostic marker in mCRC (23, 24). Both *BRAF*mut and *KRAS*mut are poor prognostic markers after liver (25–27) and lung surgery (28) and after cytoreductive surgery with hyperthermic intraperitoneal chemotherapy (29). *BRAF*mut has therefore been suggested to be one of the factors to consider before metastatic surgery. However, in the clinic, it has been observed that some patients with *BRAF*mut tumors have a relatively long survival, despite the mutational status. This was recently explored in study of 395 *BRAF*mut mCRCs, where clinical prognostic markers defined three vastly different prognostic groups (30). Our study may, at least in part, explain this as it demonstrates that CDX2 expression defines a new prognostic subgroup in patients with *BRAF*mut (53%) with a much better prognosis, comparable to wild-type patients. We also verify that CDX2 loss is a poor prognostic marker in this subgroup, as demonstrated in a very recently published study (31).

Loss of CDX2 expression also defines a smaller group (9%) of *KRAS*mut cases with a worse prognosis, but this needs to be validated due to few cases in our cohort. These results could have clinical implications when considering treatment strategies for such patients, and further studies of patients undergoing curative metastasis surgery should evaluate if CDX2 status could impact on the negative prognosis of *BRAF*mut and *KRAS*mut. CDX2 loss has also recently been demonstrated as a negative prognostic marker after liver metastasectomy, but this study did not correct for *KRAS* or *BRAF* mutation status (11). According to our results, it is a reason to believe that CDX2 loss could be a negative prognostic marker for these patients regardless of mutational status, but due to small numbers in our subgroup analysis, particularly in cases with *KRAS*mut and CDX2 loss, this needs to be verified in larger study cohorts.

Recent retrospective studies of stage II–III CRC report CDX2 loss as a potential predictor of benefit from adjuvant chemotherapy (7, 8). In the metastatic situation, the predictive effect of CDX2 loss seems rather to be the opposite (8, 9, 11). In our study, patients with CDX2 loss had poorer survival and response to chemotherapy with more often immediate disease progression on first-line combination chemotherapy, shorter PFS, and few made it to second-line treatment or secondary surgery. This might indicate that patients with CDX2 loss should have a different treatment regimen. An intuitive approach would be, as for *BRAF*mut tumors, to use triple combination therapy (32). Only a few patients were included in our subgroup analyses, however, no increased OS benefit of using doublet chemotherapy vs. monotherapy was seen in patients with CDX2 loss. It is therefore not sure that these patients will benefit from a more intensified chemotherapy regimen. Further studies on new treatment regimens in this subgroup of patients are clearly warranted, as they might need a completely different treatment approach.

The recently updated National Comprehensive Cancer Network guidelines recommend addition of *BRAF* inhibitors to standard second-line treatment in patients with *BRAF*mut tumors (33). Encouraging data have been published on EGFR-, *BRAF*-, and MEK-inhibitor combination treatment of patients with *BRAF*mut mCRC (34). However, far from all patients benefit substantially from this treatment, and it could be relevant to study if the benefit of this regimen depends on CDX2 status. Tumor MSI status is used as a predictive marker for immunotherapy effect in mCRC patients, and checkpoint inhibitors are currently recommended as second-line treatment for patients with MSI-H tumor according to the National Comprehensive Cancer Network guidelines (33). Since CDX2 loss is associated with MSI-H status and both are poor prognostic markers with poor response to standard chemotherapy in mCRC, future studies should also evaluate if the effect of checkpoint inhibitors may vary according to CDX2 status.

## Limitations

As this is a population-based study, patients with both poorer performance status, older age, and comorbidity are included, and results are therefore difficult to compare with patients included in phase III clinical trials. It is also difficult to assess the predictive effect of CDX2 in our cohort, as patients not given chemotherapy are in the worst prognostic group. Although this is a population-based study, we know that patients who did not have tumor tissue available to perform TMA, and therefore not included in the biomarker analysis, have a particularly poor prognosis (16). To remove treatment selection bias, we chose to select patients treated with first-line and first-line combination chemotherapy for further subgroup survival analyses. In some of the biomarker subgroup analyses, we therefore had few patients, which could affect the results. Our molecular analyses were mainly performed on tissue from primary tumor and not the metastatic site; however, most studies of tumor molecular alterations show high concordance between primary tumor and metastases (35). Furthermore, the evaluation of CDX2 was based on IHC analysis on small TMA sections. Although two samples were taken from each tumor, the possibility of intratumoral heterogeneity cannot be excluded. Finally, the studied patient cohort is more than 10 years old. Treatment options for mCRC patients has, however, not changed much during this time period, although today we treat more fit patients with intensified chemotherapy regimens as well as metastasectomy. There are also recent data from a Dutch population-based synchronic mCRC series reporting no difference in median OS during the past 10 years (4).

## Conclusions

In an unselected cohort of mCRC, CDX2 loss is an independent negative prognostic marker for survival. CDX2 loss indicates poor response and less survival benefits from standard chemotherapy in the metastatic situation, and effort is needed in finding new treatment regimens for this subgroup of patients. Expression of CDX2 defines a new subgroup of *BRAF*mut cases with a much better prognosis. Loss of CDX 2 also defines a smaller group of *KRAS*mut cases with a worse prognosis. CDX2 status may therefore be clinically relevant for a choice of treatment strategy.

## Data Availability Statement

The datasets generated for this study are available on request to the corresponding author.

## Ethics Statement

The studies involving human participants were reviewed and approved by Regional Committee for Medical and Health Research Ethics—REC West (Norway), Regional Ethical Committee Uppsala (Sweden) and The Regional Scientific Ethical Committees for Southern Denmark (Denmark). The patients/participants provided their written informed consent to participate in this study.

## Author Contributions

HS, PP, and BG designed the study and was responsible for patient collection. AD investigated all histological slides and was with FP, P-HE, and AM responsible for all IHC annotation. MS was responsible for the molecular analyses. KA and GE made the statistical analyses. KA drafted the manuscript with HS. All authors contributed to manuscript revision and approved the submitted version.

### Conflict of Interest

The authors declare that the research was conducted in the absence of any commercial or financial relationships that could be construed as a potential conflict of interest.
