# Supplementary material for: CDX2: A Prognostic Marker in Metastatic Colorectal Cancer Defining a Better BRAF Mutated and a Worse KRAS Mutated Subgroup
Source: Front Oncol. 2020 Feb 11;10:8. doi: 10.3389/fonc.2020.00008 (PMC7026487; doi:10.3389/fonc.2020.00008)

Supplementary Material

Table S1 1st-line palliative treatment in a population-based Scandinavian cohort of metastatic colorectal cancer patients

Treatment	All patients	CDX2 loss	CDX2 expression	p-value
5-FU + Lv	57	10 (12)	47 (13)	0.858
Irinotecan	7	2 (2)	5 (1)	0.624
5-FU + Lv + Irinotecan	52	8 (9)	44 (12)	0.468
5-FU + Lv + Oxaliplatin	168	27 (31)	141 (39)	0.217
+Cetuximab	20	4 (5)	16 (4)	1.000
+Bevacizumab	21	2 (2)	19(5)	0.282

Abbreviations: FU: Fluorouracil; Lv: Leucovorin; p-value: chi-square test.

Table S2 Results from multiple logistic regression of CDX2 status in a population-based Scandinavian cohort of metastatic colorectal cancer patients

	Unadjusted models				Fully adjusted model (n = 408)			Simplified model[a)] (n = 433)		
Predictor variables	n	OR	95 % CI	p	OR	95 % CI	p	OR	95 % CI	p
Female	452	1.40	(0.88, 2.25)	0.158	1.04	(0.57, 1.91)	0.891			
Elderly (> 75 years)	452	1.08	(0.66, 1.76)	0.770	1.02	(0.54, 1.93)	0.960			
Right-sided tumour	445	2.77	(1.71, 4.48)	<0.001	1.15	(0.59, 2.26)	0.678			
Tumour grade 3	437	4.19	(2.50, 7.03)	<0.001	2.17	(1.12, 4.21)	0.023	2.67	1.48, 4.82	0.001
Liver metastases	452	0.69	(0.43, 1.11)	0.122	1.29	(0.67, 2.45)	0.442			
Lung metastases	452	0.52	(0.28, 0.96)	0.027	0.78	(0.35, 1.63)	0.472			
Peritoneal metastases	452	1.66	(0.96, 2.87)	0.076	1.42	(0.69, 2.92)	0.345			
Lymph node metastases	452	2.13	(1.31, 3.47)	0.003	1.42	(0.75, 2.69)	0.282			
KRAS mutation	437	0.27	(0.15, 0.48)	<0.001	0.71	(0.32, 1.56)	0.389			
BRAF mutation	448	10.27	(6.03, 17.47)	<0.001	7.22	(3.34, 15.61)	<0.001	8.88	5.06, 15.59	< 0.001
MSI-H	441	8.82	(4.25, 18.31)	<0.001	1.66	(0.64, 4.32)	0.297			

Abbreviations: Right-sided tumour: site of colon cancer in ascending colon and transversum; MSI-H: microsatellite instable high; OR: odds ratio; CI: confidence interval; p: likelihood ratio test
[a)] from backward stepwise selection at nominal significance level 0.05.

Table S3 Response after 1st- and 2nd-line chemotherapy in patients with CDX2 status and response registered in a population-based Scandinavian cohort of metastatic colorectal cancer patients

Treatment	Response	All patients	CDX2 -	CDX2 +	p-value
1st-line chemotherapy	CR/PR	102 (41)	14 (35)	88 (43)	0.011
	SD	100 (41)	12 (30)	88 (43)	
	PD	45 (18)	14 (35)	31 (15)	
1st-line combination chemotherapy	CR/PR	90 (46)	10 (35)	80 (49)	0.003
	SD	77 (40)	9 (31)	68 (41)	
	PD	27 (14)	10 (35)	17 (10)	
2nd-line chemotherapy	CR/PR	25 (17)	3 (17)	22 (17)	0.959
	SD	71 (47)	8 (44)	63 (48)	
	PD	54 (36)	7 (39)	47 (36)	

Abbreviations: CDX2-: CDX2 loss; CDX2+: CDX2 expression; CR: complete response; PR: partial response; SD: stable disease; PD: progressive disease; p-value: chi-square test.

Figure S1 Median overall survival (OS) and progression-free survival (PFS) in a population-based Scandinavian cohort of metastatic colorectal cancer according to CDX2, *BRAF* and *KRAS* status. Kaplan-Meier curves were calculated with log-rank test for p-value. A) Median OS in all patients regardless of treatment given was 14 months if double wildtype (n=165, e=157), 13 months if *KRAS*mut/CDX2 expression (n=163, e=156) vs 2 months if *KRAS*mut/CDX2 loss (n=16, e=16) (p < 0.001) and 10 months if *BRAF*mut/CDX2 expression (n=45, e=44) vs 6 months if *BRAF*mut/CDX2 loss (n=51, e=51) (p = 0.008). B) Median OS in patients given 1st-line chemotherapy was 24 months if double wildtype (n=110, e=102), 20 months if *KRAS*mut/CDX2 expression (n=105, e=99) vs 9 months if *KRAS*mut/CDX2 loss (n=6, e=6) (p = 0.002) and 15 months if *BRAF*mut/CDX2 expression (n=30, e=29) vs 11 months if *BRAF*mut/CDX2 loss (n=25, e=25) (p = 0.029). C) Median PFS in patients given 1st-line chemotherapy was 9 months if double wildtype (n=110, e=107), 8 months if *KRAS*mut/CDX2 expression (n=105, e=99) vs 2 months if *KRAS*mut/CDX2 loss (n=6, e=6) (p = 0.025) and 8 months if *BRAF*mut/CDX2 expression (n=30, e=29)vs 5 months if *BRAF*mut/CDX2 loss (n=24, e=24) (p = 0.031). *Abbreviations:* double wildtype: *BRAF* and *KRAS* wildtype; *BRAF*mut*: BRAF* mutation*; KRAS*mut*: KRAS* mutation; CDX2- : CDX2 loss; CDX2+: CDX2 expression; n: number; e: events.

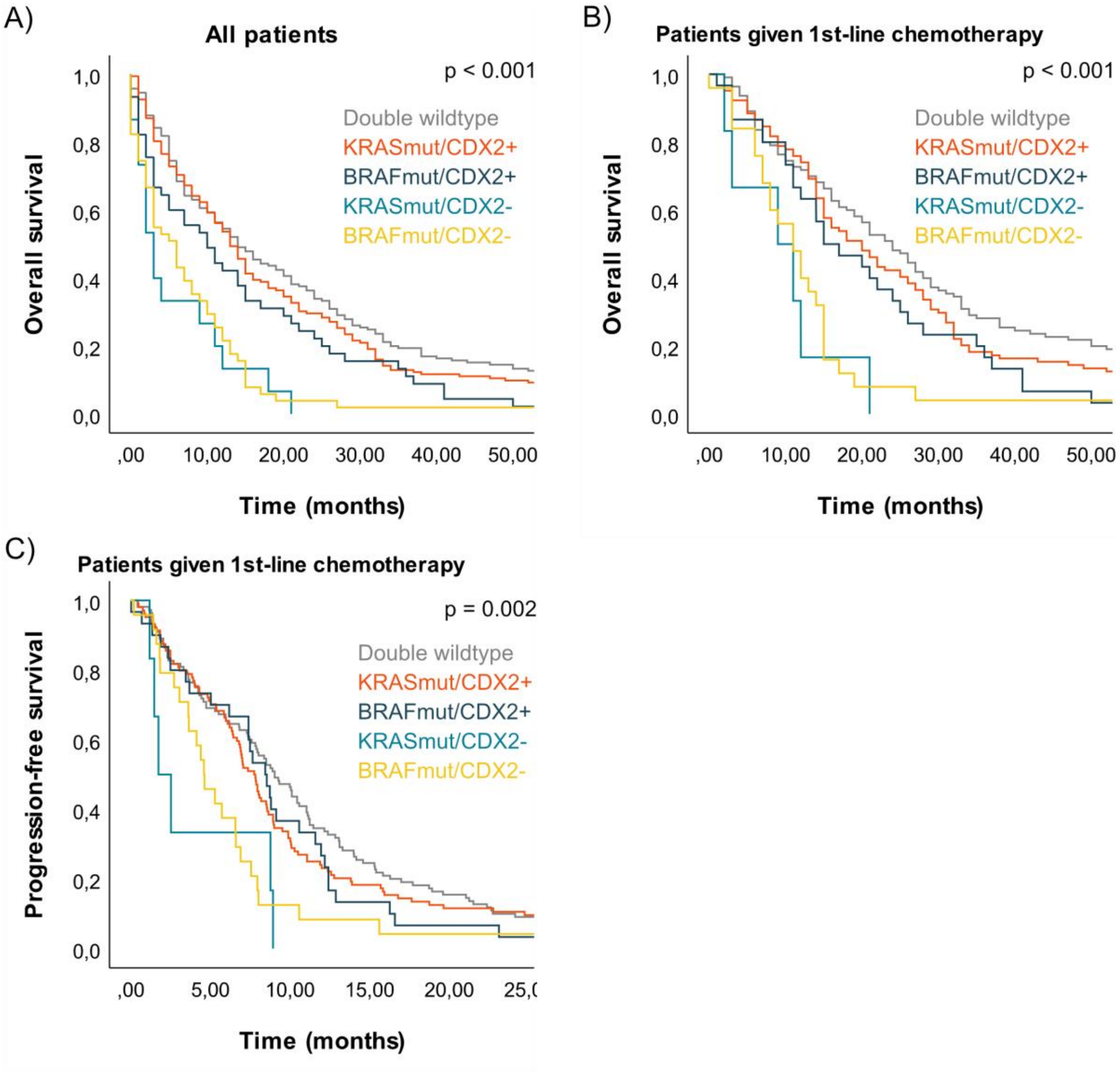

Figure S2 Median overall survival (OS) in a population-based Scandinavian cohort of metastatic colorectal cancer patients given 1st-line chemotherapy according to CDX2 status. Kaplan-Meier curves was calculated with log-rank test for p-value and univariate Cox regression for HR and 95% CI. A) Median OS in *BRAF* wildtype patients B) Median OS in *BRAF* mutated patients C) Median OS in *KRAS* wildtype patients D) Median OS in *KRAS* mutated patients E) Median OS in microsatellite stable (MSS) patients F) Median OS in microsatellite instable high (MSI-H)
Abbreviations: n: number; e: events; HR: Hazard ratio; CI: confidence interval.

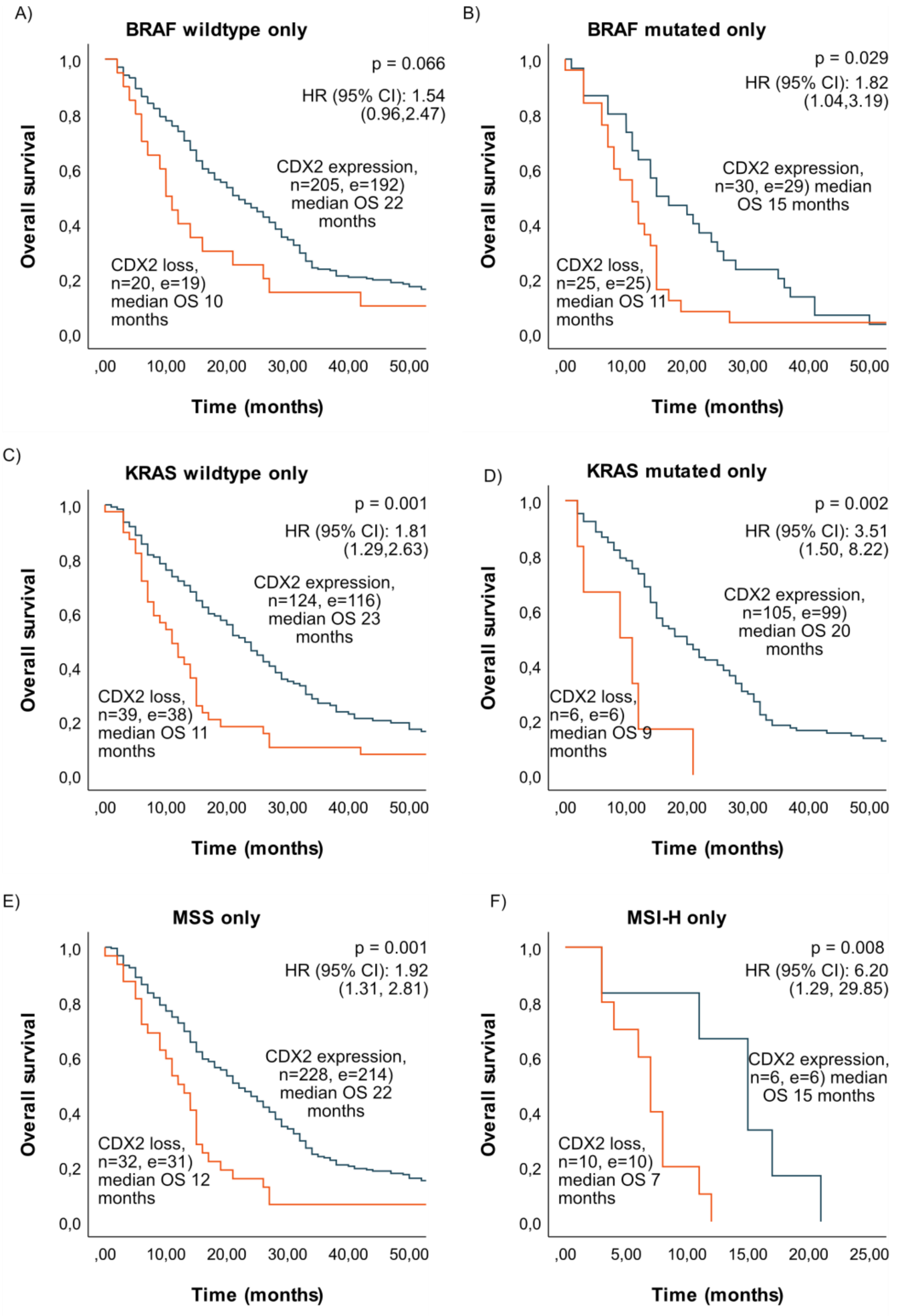

Supplement: Supplementary file 1 [file Data_Sheet_1.PDF]
